# Disparities in Peripheral Artery Disease Hospitalizations Identified Among Understudied Race-Ethnicity Groups

**DOI:** 10.3389/fcvm.2021.692236

**Published:** 2021-05-24

**Authors:** LaiTe Chen, Donglan Zhang, Lu Shi, Corey A. Kalbaugh

**Affiliations:** ^1^Department of Cardiology, Sir Run Run Shaw Hospital, School of Medicine, Zhejiang University, Hangzhou, China; ^2^Department of Health Policy and Management, University of Georgia, Athens, GA, United States; ^3^Department of Public Health Sciences, Clemson University, Clemson, SC, United States

**Keywords:** peripheral artery disease, chronic limb threatened ischemia, disparities, medical expenditure, mortality

## Abstract

**Background:** To assess racial/ethnic differences in disease severity, hospital outcomes, length of stay and healthcare costs among hospitalized patients with peripheral artery disease (PAD).

**Methods:** This study used data from the National Inpatient Sample (NIS) to explore the racial/ethnic disparities in PAD-related hospitalizations including presence of PAD with chronic limb threatened ischemia (CLI), amputation, in-hospital mortality, length of hospital stays and estimated medical costs. Race-ethnicity groups included non-Hispanic White, Black, Hispanic, Asian or Pacific Islander, Native American, and others (multiple races). Regression analyses adjusted for age, gender, Charlson Comorbidity Index, primary payer, patient location, bed size of the admission hospital, geographic region of the hospital, and rural/urban location of the hospital.

**Results:** A total of 341,480 PAD hospitalizations were identified. Compared with non-Hispanic Whites, Native Americans had the highest odds of PAD with CLI (OR = 1.77, 95% CI: 1.61, 1.95); Black (OR = 1.71, 95% CI: 1.66, 1.76) and Hispanic (OR = 1.36, 95% CI: 1.31,1.41) patients had higher odds of amputation; Asian or Pacific Islanders had a higher mortality (OR = 1.20, 95% CI: 1.01,1.43), whereas Black (OR = 0.81, 95% CI: 0.76, 0.87) patients has a lower mortality; Asian or Pacific Islanders incurred higher overall inpatient costs (Margin = 30093.01, 95% CI: 28827.55, 31358.48) and most prolonged length of stay (IRR = 0.14, 95% CI: 0.09, 0.18).

**Conclusions:** Our study identified elevated odds of amputation among Hispanic patients hospitalized with PAD as well as higher hospital mortality and medical expenses among Asian or Pacific Islander PAD inpatients. These two demographic groups were previously thought to have a lower risk for PAD and represent important populations for further investigation.

## Introduction

Peripheral artery disease (PAD) is an atherosclerotic disease that can lead to increased risk of functional limitations (1), reduction in quality of life and death (2). In the United States, there were 199,423 deaths due to PAD recorded between 1980 and 2014 with increasing age-adjusted mortality rates (3) and clear patterns of socioeconomic inequality (4). From the perspective of healthcare cost containment, PAD-related hospitalizations are expensive (5) and represent a growing problem in the United States (6).

There is substantial evidence of a higher prevalence of hospitalized PAD among Blacks than Whites (7–10) and Native Americans also have high prevalence of PAD as compared with non-Hispanic Whites (11). However, there is limited information on the burden of PAD among other racial/ethnic minority groups (12), especially among the PAD inpatients (13). While a study using the population-representative sample of National Health and Nutrition Examination and Survey (NHANES) found Mexican-American women had a higher prevalence of PAD than non-Hispanic white women (14), other data sources do not show this pattern of Hispanic disadvantage (15). A review of the literature actually suggests that the “Hispanic paradox” in cardiovascular health–where Hispanic Americans have lower prevalence of coronary heart disease but higher burden of cardiovascular risk factors–may also exist for arteries in the extremities and neck (16). Knowing that Hispanic Americans are at elevated risk for diabetes (17) and the link between diabetes and PAD (18), this “Hispanic paradox” hypothesis in PAD needs further validation. Given the growing population sizes of Hispanic Americans and Asian Americans (19), it is important to understand the possible disparity patterns of these demographic groups, and the costly nature of PAD hospitalization indicates that it is important to study the racial/ethnic patterns in PAD hospitalizations.

In this study, we aimed to study the racial/ethnic patterns in PAD hospitalizations, including presentation of critical limb ischemia (CLI), PAD-related amputations, in-patient mortality, length of stay, and expenditures. To accomplish these aims we used data from the Nationwide Inpatient Sample (NIS) over the period of 2011 through 2015.

## Methods

### Study Population

The present study is a cross-sectional analysis with 5 years (2011–2015) of NIS data including inpatients with a PAD-related diagnostic or procedure code. NIS is the largest inpatient care database developed for the Healthcare Cost and Utilization Project (HCUP) (20) in the United States, which is suitable for developing national estimates and analyses for hospitalizations due to specific diseases (21, 22). Data are taken from discharge abstracts and include information on demographic characteristics, diagnostic/procedure codes, dates of admission and discharge, hospital bed size, hospital location, teaching status, and medical charges. Any sample with missing data on race was excluded which counted for 7.8% of whole population.

### Measurement of PAD

Relevant data were measured using the International Classification of Disease, 9th revision, Clinical Modification (ICD-9-CM; 2011–2015). PAD [General PAD and PAD with CLI (23)] and major lower limb amputation were identified with ICD-9 codes (Supplementary Table 1). As opposing to PAD with CLI, general PAD was defined as PAD without CLI.

### Demographic, Comorbidity Measures, and Socioeconomic Characteristics

Demographic variables included age, race and sex. HCUP coding includes race and ethnicity in one data element (RACE) and is categorized as non-Hispanic White, Black, Hispanic, Asian or Pacific Islander, Native American, and Others (multiple races) (24). Clinical characteristics included the Charlson comorbidity index (CCI) (25, 26), bed size, rural/urban location and region of the hospital. Patients with CCI ≥3 were considered as high risk. Socioeconomic characteristics included primary payer (Medicare, Medicaid, Private insurance, Self-pay, No charge, Other) and the use of emergency department services (Central, counties of metro areas of ≥1 million population; Fringe, counties of metro areas of ≥1 million population; Counties in metro areas of 250,000–999,999 population; Counties in metro areas of 50,000–249,999 population; Micropolitan counties; Not metropolitan or micropolitan counties).

### Hospitalization Measures

“Outcomes” included proportion of inpatients with PAD and CLI, performance of major lower limb amputation, mortality during hospitalization, length of hospital stay, and medical cost. To acquire a closer assessment of the medical expenses, the cost-to-charge ratios provided by the Agency of Healthcare Research and Quality was used to convert hospital charges into estimated payments paid by insurance and patients. All outcomes were presented in strata of race.

### Statistical Analysis

Categorical variables were presented as proportions and the chi-square test was performed to screen for statistical differences across subgroups. Continuous variables were presented in mean ± standard deviation (SD, normally distributed) and median (InterQuartile Range, IQR; non-normal distribution). ANOVA test was used to identify statistical differences on normally distributed data whereas the Kruskal-Wallis test was applied to the non-normally distributed data.

To illustrate the racial/ethnic distribution (reference = non-Hispanic White) of PAD with CLI, multivariate logistic regression modeling was performed to study amputation and in-hospital mortality, negative binomial regression was applied to evaluate the length of hospital stay and generalized linear model with log link and gamma distribution was used to estimate medical costs. In each model, the racial distribution for each outcome was adjusted by age, sex, CCI, primary payer, emergency department services record, bed size of the admitted hospital, region of the hospital, and location of the hospital. Sampling weights were adjusted in all analyses. Sampling weights were used in all descriptive and regression analyses. Data were analyzed using Stata 15 (Stata Corp, College Station, TX), and the threshold of statistical significance was set at α = 0.05.

## Results

### Overall Demographic and Clinical Characteristics (Table 1)

We identified a total of 341,480 PAD hospitalizations. Over half of the inpatients were male (average 55.1% per year) and the average age was 62.6 (SD: 19.0). Regarding ethnicity, 65.3% of hospitalizations were among non-Hispanic White patients, 19.5% were among Black patients while Asian or Pacific Islander- and Native American- patient hospitalizations constituted 1.6 and 0.7%. Over forty percent of the hospitalizations were for patients considered high risk (average 45.3% per year) (Table 1).

**Table 1:** Demographic and Hospitalization Measures by race-ethnicity group.

	**Overall**	**White**	**Black**	**Hispanic**	**Asian or Pacific islander**	**Native american**	**Other**
	**(*N* = 341,480)**	**(*N* = 222,834)**	**(*N* = 66,725)**	**(*N* = 34,538)**	**(*N* = 5,459)**	**(*N* = 2,226)**	**(*N* = 9,698)**
Age, mean (SD)	62.6 (19.0)	64.7 (17.8)	59.0 (18.9)	57.4 (22.2)	60.2 (23.6)	58.5 (18.1)	59.2 (22.2)
Sex, % Female	153,447 (44.9%)	96,888 (43.5%)	33,294 (49.9%)	15,447 (44.7%)	2,575 (47.2%)	1,013 (45.5%)	4,230 (43.6%)
CCI							
Moderate risk	186,640 (54.7%)	130,157 (58.4%)	31,872 (47.8%)	15,738 (45.6%)	2,512 (46.0%)	1,045 (46.9%)	5,316 (54.8%)
High risk	154,840 (45.3%)	92,677 (41.6%)	34,853 (52.2%)	18,800 (54.4%)	2,947 (54.0%)	1,181 (53.1%)	4,382 (45.2%)
PAD							
General PAD	181,703 (53.2%)	123,938 (55.6%)	32,892 (49.3%)	15,666 (45.4%)	3,028 (55.5%)	1,026 (46.1%)	5,153 (53.1%)
PAD with CLI	159,777 (46.8%)	98,896 (44.4%)	33,833 (50.7%)	18,872 (54.6%)	2,431 (44.5%)	1,200 (53.9%)	4,545 (46.9%)
Major amputation	47,617 (13.9%)	27,585 (12.4%)	12,297 (18.4%)	5,439 (15.7%)	732 (13.4%)	395 (17.7%)	1,169 (12.1%)
In-Hospital mortality	8,691 (2.5%)	5,937 (2.7%)	1,483 (2.2%)	763 (2.2%)	181 (3.3%)	48 (2.2%)	279 (2.9%)
LOS, median (IQR)	5 (7)	4.0 (6.0)	6.0 (7.0)	5.0 (7.0)	4.0 (7.0)	5.0 (6.0)	5.0 (7.0)
Cost ($), median (IQR)	15180.3 (18963.4)	15238.6 (18636.0)	15043.2 (18911.0)	14618.7 (19756.4)	17000.1 (24677.4)	15154.6 (19716.5)	15796.0 (21271.0)

*SD, standard deviation; CCI, Charlson Comorbidity Index; PAD, peripheral artery disease; CLTI, chronic limb threatened ischemia; LOS, length of stay; IQR, interquartile range*.

### Demographics and Clinical Characteristics, by Race-Ethnic Groups (Table 1)

The average age of non-Hispanic White and Asian or Pacific Islander patients were 64.7 (SD: 17.8) and 60.2 (SD: 23.6), respectively. Over half of patients were male. Less than half of patients were categorized as high risk in non-Hispanic White patients (41.6%) whereas over half of patients in the other ethnic groups were high risk (Table 1).

### Presentation of PAD With CLI (Table 2)

Fourty-six point eight percent of the hospitalized patients with PAD had CLI. Compared with non-Hispanic White patients, other ethnicities (including Black, Hispanic, and Native American) had higher odds of CLI. Native American patients had the highest odds of CLI (OR = 1.77, 95% CI: 1.61, 1.95; Table 2).

**Table 2:** Multivariate analysis of PAD with CLI, by race-ethnicity group.

	**OR (95% CI)**	***p*-value**
Race-Ethnicity group^a^		
Black	1.42 (1.39, 1.45)	<0.0001
Hispanic	1.55 (1.51, 1.59)	<0.0001
Asian or Pacific islander	0.88 (0.82, 0.94)	<0.0001
Native american	1.77 (1.61, 1.95)	<0.0001
Other	1.10 (1.05, 1.16)	<0.0001

*PAD, peripheral artery disease; CLTI, chronic limb threatened ischemia; OR, Odds Ratio; CI, Confidence Interval*.

a *Referent is non-white Hispanics*.

### Presentation of Amputation at Hospitalization (Table 3)

Over 10% of patients with PAD received major lower limb amputation (average: 13.9% per year). Among different ethnicities, non-Hispanic White patients had the lowest proportion of cases that receiving major amputation whereas Black (OR = 1.71, 95% CI: 1.66, 1.76), Hispanic (OR = 1.36, 95% CI: 1.31, 1.41), and Native American (OR = 1.48, 95% CI: 1.31, 1.67) patients had higher odds of undergoing amputation. No statistical difference was found between non-Hispanic White and Asian or Pacific Islander patients (Table 3).

**Table 3:** Multivariate analysis of major limb amputation, by race-ethnicity group.

	**OR (95% CI)**	***p*-value**
Race-Ethnicity group^a^		
Black	1.71 (1.66, 1.76)	<0.0001
Hispanic	1.36 (1.31, 1.41)	<0.0001
Asian or Pacific islander	0.93 (0.85, 1.03)	0.18
Native american	1.48 (1.31, 1.67)	<0.0001
Other	1.06 (0.99, 1.14)	0.12

*OR, Odds Ratio; CI, Confidence Interval*.

a *Referent is non-white Hispanics*.

### In-hospital Mortality (Table 4)

Less than 3% of patients with PAD died in the hospital (average: 2.5%). Compared with non-Hispanic White patients, Asian or Pacific Islander had higher odds of in-hospital death (in average OR = 1.20, 95% CI: [1.01, 1.43]) whereas Black (OR = 0.81, 95% CI: 0.76, 0.87) and Hispanic (OR = 0.84, 95% CI: 0.77, 0.92) patients had lower odds of in-hospital death. The difference of in-hospital mortality between Native American and non-Hispanic White patients was not statistically significant (Table 4).

**Table 4:** Multivariate analysis of in-hospital mortality, by race-ethnicity group.

	**OR (95% CI)**	***p*-value**
Race-Ethnicity group^a^		
Black	0.81 (0.76, 0.87)	<0.0001
Hispanic	0.84 (0.77, 0.92)	<0.0001
Asian or Pacific islander	1.20 (1.01, 1.43)	0.04
Native american	0.75 (0.54, 1.04)	0.09
Other	1.14 (0.99, 1.30)	0.06

*OR, Odds Ratio; CI, Confidence Interval*.

a *Referent is non-white Hispanics*.

### Length of Stay (Table 5)

The average length of hospital stays for patients with PAD was 5 (IQR: 7) days. Compared to non-Hispanic Whites, a prolonged length of stay was observed in Black (IRR = 0.12, 95% CI: 0.11, 0.13), Asian or Pacific Islander (IRR = 0.14, 95% CI: 0.09, 0.18) and Hispanic (IRR = 0.09, 95% CI: 0.07, 0.11) patients. Native American and non-Hispanic White patients had similar length of stays (Table 5).

**Table 5:** Multivariate analysis of length of stay, by race-ethnicity group.

	**IRR (95% CI)**	***p*-value**
Race-Ethnicity group^a^		
Black	0.12 (0.11, 0.13)	<0.0001
Hispanic	0.09 (0.07, 0.11)	<0.0001
Asian or Pacific islander	0.14 (0.09, 0.18)	<0.0001
Native american	0.03 (−0.02, 0.08)	0.21
Other	0.09 (0.06, 0.12)	<0.0001

*IRR, Incidence Rate Ratio; CI, Confidence Interval*.

a *Referent is non-white Hispanics*.

### Medical Expenditures (Table 6)

The average medical expenses for hospitalized patients with PAD was $15,180.3 (IQR: $18,963.4), after being converted from charges to medical costs. Compared with non-Hispanic White patients (Margin = 25231.59, 95% CI: 25091.87, 25371.32), more medical expenses were expected in Asian or Pacific Islander patients (Margin = 30093.01, 95% CI: 28827.55, 31358.48) whereas less costs were estimated with Black, Hispanic, and Native American patients. Among racial-ethnic groups, the least economic burden was carried by Native American patients (Margin = 22240.41, 95% CI: 21167.21, 23313.61) whereas the cost of Hispanic patients were similar to non-Hispanic White's (Margin = 24568.83, 95% CI: 24164.78, 24972.88) (Table 6).

**Table 6:** Marginal effect analysis of cost, by race-ethnicity group.

	**Margin**	**95% CI**
Race-Ethnicity group		
non-White Hispanics	25231.59	(25091.87, 25371.32)
Black	24307.54	(24052.49, 24562.59)
Hispanic	24568.83	(24164.78, 24972.88)
Asian or Pacific islander	30093.01	(28827.55, 31358.48)
Native american	22240.41	(21167.21, 23313.61)
Other	28389.73	(27492.14, 29287.32)

*CI, Confidence Interval*.

## Discussion

In the present study, we sought to study the racial/ethnic patterns in PAD hospitalizations. Among hospitalized patients with PAD, we identified a significantly higher in-hospital mortality among Asian American inpatients with PAD, presenting an aspect of PAD risk that had not previously been observed. We further observed a higher percentage of critical limb ischemia in Black and Hispanic patients vs. non-Hispanic White patients. A higher proportion of Black and Hispanic patients also received major amputations during their hospitalizations. While this pattern of elevated amputation risk has been well-documented among Black inpatients with PAD, our study represents an important contribution regarding the heightened risks of poor limb outcomes to which Hispanic patients are subject (22, 27).

Asian Americans (especially female Asian Americans) were previously thought to have a lower prevalence of PAD as compared with non-Hispanic Whites (28). Hispanic people in the United States were also found to have a much lower incidence of PAD than non-Hispanic Whites (16, 29) and it has been suggested that the “Hispanic paradox” as observed in heart disease might also exist for PAD (16). Our study of the nationwide sample of PAD inpatients serves an important piece of evidence that we need to be cautious about the interpretation of cardiovascular health “advantages” among Hispanics for PAD risk. From the perspective of severity of the disease and the performance of end-stage procedures, we actually observed worse complications among Hispanic inpatients. This finding sheds a light in epidemiological study of PAD across racial/ethnic groups: certain racial/ethnic groups' seemingly lower PAD incidence in previous analysis might result from the possible under-diagnosis of their PAD status at an early stage. There has been evidence against the “Hispanic paradox” based upon longitudinal data on cardiovascular mortality (US-born Mexican Americans had higher risk than Whites while Mexico-born people had similar risk with Whites) (30). It is plausible that the observed low PAD incidence among Hispanic Americans in previous studies could at least partially be a function of low detection rate given the high rate of being uninsured among this population (31).

Previous studies have demonstrated that advanced age was a major risk factor for PAD (32, 33). However, Hispanic and Native American patients were relatively young in the present study, indicating the possibility that PAD might affect these minority groups at a younger age than the non-Hispanic Whites. More Hispanic and Native American patients presented with worse disease (CLI) than non-Hispanic White patients, suggesting that the issue of under-diagnosis and under-treatment in early disease stages might be more serious among non-White minority groups than among non-Hispanic Whites.

Peripheral artery disease has a well-established high risk of death following diagnosis (34). Death rates are particularly high among patients diagnosed with PAD in the inpatient setting (35). Less is known about the racial/ethnic pattern of in-hospital mortality among PAD patients, particularly in patients that are not Black or White. A recent vascular intervention registry-based study found better short-term survival among Black and Hispanic patients compared to White patients (36). Our study supports the survival advantage among these two minority groups and is a potentially surprising finding of improved short-term survival despite poor short-term limb outcomes in Black and Hispanic patients compared to White patients. Conversely, and equally surprising, we also found that Asian or Pacific Islander patients had higher mortality than other ethnic groups. These findings warrant further examination of strategies that are preventing poor limb outcomes in Whites and Asian/Pacific Islanders and those that are preventing early death among Blacks and Hispanics. Patients with mild condition of PAD may require a revascularization instead of amputation. Among those patients, the in-hospital mortality may be affected not only by ethnicities but also by the types of procedure (such as endovascular intervention vs. bypass surgery).

For the length of hospital stay, compared with non-Hispanic Whites, Asian or Pacific Islander and Hispanic patients had a prolonged hospital stay, which might indicate a more complicated state of the disease. For medical expenses, Asian or Pacific Islander patients paid the highest medical expenses among racial-ethnic groups, which could also indicate a more complicated hospitalization case among these Asian/Pacific Islander patients. Poor socioeconomic status may lead to underreporting and may contribute to the development of PAD in similar conditions (37–39). It is plausible that demographic groups with less affordable medical care in the early stage of the disease were more likely to have critical cases at the hospitalization point, which was what we observed in our current study and was consistent with the patterns identified in previous studies (40, 41).

Our study has several strengths, including identification of a contemporary population of patients with PAD. The impact of coding errors is reduced by the large sample size of our data source. We applied weights to account for the sampling shift, thus the results represent the outcomes among the national inpatient population with PAD. Our study examined PAD severity and demonstrated disparities by groups, which provides a new perspective to the optimization of treating early-stage PAD among ethnicities.

There were several limitations in the present study. First, the datasets analyzed in this study only contained patients who required inpatient medical care (emergency medical service not included). Claims billed in outpatient clinics and private pharmacies were not included, as such the total prevalence of PAD was not estimable. Thus, our findings are only generalizable to those with disease severe enough to require a hospitalization. Second, race was missing in a proportion of our sample and we chose not to impute this data, which may bias our estimates. However, our robust sample size allowed us to have precise estimates at all outcomes and we do not believe this to be a limitation that would alter our conclusions. Third, other health care use outcomes such as medications were not acquired from the datasets that may affect our estimates for the outcome. However, considering the target population was set as the patients who required emergency medical attention and/or received the intensive procedure such as amputations, the impact of missing medication information on the outcome estimates of such critical patients were relatively small. Fourth, the NIS sampling strategy changed in 2012, which we accounted for by adjusting using sampling weights. Fifth, charging practices are based on administrative policy. Various coding systems may be adopted by different hospitals. However, the adjustments of hospital volume, location, and teaching status were performed, assuming hospitals with same levels of stratifications had similar billing regulations. Seventh, there were other potential confounders such as education level and annual income which may affect the association. However, these factors are not recorded in the NIS database. Lastly, PAD related data from the US Nationwide Emergency Department Sample (NEDS) were not included in this study due to the deployment of different clinical strategies, medical resource, and healthcare insurance coverage, which were more appropriately to be presented in further study.

## Conclusions

The racial/ethnic disparity pattern in PAD is much broader than the Black-White difference, which has been the focus and paradigm of PAD disparity research in the U.S (5, 42). We observed race and ethnicity-related disparities regarding the severity of PAD at hospitalization and outcomes of PAD during the hospitalization, especially in-hospital mortality among two demographic groups (Hispanics and Asian/Pacific Islanders) previously thought to be at lower risk for PAD. These racial disparities indicate the importance of PAD prevention, early detection and management work among the racial/ethnic minority groups that traditionally received less research attention. These findings also speak more broadly to general issues of healthcare coverage and equity that warrant further study in patients with PAD.

## Data Availability Statement

The datasets generated for this study can be found in online repositories. The names of the repository/repositories and accession number(s) can be found in the article/Supplementary Material.

## Author Contributions

LC: writing-original draft preparation, software, and methodology. DZ: data curation, software, writing-reviewing, and editing. LS: conceptualization, supervision, writing-reviewing, and editing. CK: conceptualization, supervision, writing-reviewing, and editing. All authors contributed to the article and approved the submitted version.

## Conflict of Interest

The authors declare that the research was conducted in the absence of any commercial or financial relationships that could be construed as a potential conflict of interest.

## References

[1] Iwamoto A Kajikawa M Maruhashi T Iwamoto Y Oda N Kishimoto S. Vascular function and intima-media thickness of a leg artery in peripheral artery disease: a comparison of buerger disease and atherosclerotic peripheral artery disease. J Atheroscler Thromb. (2016) 23:1261–9. 10.5551/jat.35436 27169920 PMC5113743

[2] Fowkes FG Aboyans V Fowkes FJ McDermott MM Sampson UK Criqui MH. Peripheral artery disease: epidemiology and global perspectives. Nat Rev Cardiol. (2017) 14:156–70. 10.1038/nrcardio.2016.179 27853158

[3] Roth GA Dwyer-Lindgren L Bertozzi-Villa A Stubbs RW Morozoff C Naghavi M. Trends and patterns of geographic variation in cardiovascular mortality among us counties, 1980-2014. JAMA. (2017) 317:1976–92. 10.1001/jama.2017.4150 28510678 PMC5598768

[4] Pande RL Creager MA. Socioeconomic inequality and peripheral artery disease prevalence in us adults. Circulation. (2014) 7:532–9. 10.1161/CIRCOUTCOMES.113.000618 24987053 PMC4219271

[5] Reis JP Michos ED von Mühlen D Miller ER III. Differences in vitamin d status as a possible contributor to the racial disparity in peripheral arterial disease. Am J Clin Nutr. (2008) 88:1469–77. 10.3945/ajcn.2008.26447 19064505

[6] Kalbaugh CA Kucharska-Newton A Wruck L Lund JL Selvin E Matsushita K. Peripheral artery disease prevalence and incidence estimated from both outpatient and inpatient settings among medicare fee-for-service beneficiaries in the atherosclerosis risk in communities (aric) study. J Am Heart Assoc. (2017) 6:e003796. 10.1161/JAHA.116.003796 28468784 PMC5524052

[7] Arya S Binney Z Khakharia A Brewster LP Goodney P Patzer R. Race and socioeconomic status independently affect risk of major amputation in peripheral artery disease. J Am Heart Assoc. (2018) 7:e007425. 10.1161/JAHA.117.007425 29330260 PMC5850162

[8] Matsushita K Sang Y Ning H Ballew SH Chow EK Grams ME. Lifetime risk of lower-extremity peripheral artery disease defined by ankle-brachial index in the united states. J Am Heart Assoc. (2019) 8:e012177. 10.1161/JAHA.119.012177 31500474 PMC6818002

[9] Smith SL Matthews EO Moxon JV Golledge J. A systematic review and meta-analysis of risk factors for and incidence of 30-day readmission after revascularization for peripheral artery disease. J Vasc Surg. (2019) 70:996–1006.e1007. 10.1016/j.jvs.2019.01.079 31445653

[10] McDermott MM Polonsky TS Kibbe MR Tian L Zhao L Pearce WH. Racial differences in functional decline in peripheral artery disease and associations with socioeconomic status and education. J Vasc Surg. (2017) 66:826–34. 10.1016/j.jvs.2017.02.037 28502539 PMC5621652

[11] Baxter AR Jacobowitz GR Guo Y Maldonado T Adelman MA Berger JS. Increased prevalence of moderate and severe peripheral arterial disease in the american indian (ai)/alaskan native (an) population; a study of 96,000 ai/an. Ann Vasc Surg. (2017) 38:177–83. 10.1016/j.avsg.2016.08.002 27554686

[12] Allison MA Gonzalez F Raij L Kaplan R Ostfeld RJ Pattany MS. Cuban Americans have the highest rates of peripheral arterial disease in diverse Hispanic/Latino communities. J Vasc Surg. (2015) 62:665–72. 10.1016/j.jvs.2015.03.065 26141696 PMC4550525

[13] Abola MTB Golledge J Miyata T Rha SW Yan BP Dy TC. Asia-pacific consensus statement on the management of peripheral artery disease: a report from the Asian pacific society of atherosclerosis and vascular disease Asia-pacific peripheral artery disease consensus statement project committee. J Atheroscler Thromb. (2020) 27:809–907. 10.5551/jat.53660 32624554 PMC7458790

[14] Ostchega Y Paulose-Ram R Dillon CF Gu Q Hughes JP. Prevalence of peripheral arterial disease and risk factors in persons aged 60 and older: data from the national health and nutrition examination survey 1999–2004. J Am Geriatr Soc. (2007) 55:583–9. 10.1111/j.1532-5415.2007.01123.x 17397438

[15] Collins TC Petersen NJ Suarez-Almazor M Ashton CM. The prevalence of peripheral arterial disease in a racially diverse population. Arch Intern Med. (2003) 163:1469–74. 10.1001/archinte.163.12.1469 12824097

[16] Forbang NI Hughes-Austin JM Allison MA Criqui MH. Peripheral artery disease and non-coronary atherosclerosis in Hispanics: another paradox? Prog Cardiovasc Dis. (2014) 57:237–43. 10.1016/j.pcad.2014.07.008 25443822 PMC4254403

[17] McBean AM Li S Gilbertson DT Collins AJ. Differences in diabetes prevalence, incidence, and mortality among the elderly of four racial/ethnic groups: Whites, blacks, Hispanics, and Asians. Diabetes Care. (2004) 27:2317–24. 10.2337/diacare.27.10.2317 15451894

[18] Britton KA Mukamal KJ Ix JH Siscovick DS Newman AB de Boer IH. Insulin resistance and incident peripheral artery disease in the cardiovascular health study. Vasc Med. (2012) 17:85–93. 10.1177/1358863X11436195 22402937 PMC3563259

[19] Frey WH. Diversity Explosion: How New Racial Demographics are Remaking America. Charlotte, NC: Brookings Institution Press (2018).

[20] Rockville M. Hcup national inpatient sample (nis). Healthcare Cost and Utilization Project (HCUP): Agency for Healthcare Research and Quality (2012). Available online at: www.hcup-us.ahrq.gov/nisoverview.jsp (accessed October 6, 2020).

[21] Rowe VL Lee W Weaver FA Etzioni D. Patterns of treatment for peripheral arterial disease in the united states: 1996-2005. J Vasc Surg. (2009) 49:910–7. 10.1016/j.jvs.2008.11.054 19341885

[22] Rowe VL Weaver FA Lane JS Etzioni DA. Racial and ethnic differences in patterns of treatment for acute peripheral arterial disease in the United States, 1998-2006. J Vasc Surg. (2010) 51:S21–6. 10.1016/j.jvs.2009.09.066 20080006

[23] Agarwal S Sud K Shishehbor MH. Nationwide trends of hospital admission and outcomes among critical limb ischemia patients: from 2003-2011. J Am Coll Cardiol. (2016) 67:1901–13. 10.1016/j.jacc.2016.02.040 27012780

[24] Monlezun DJ Lawless S Palaskas N Peerbhai S Charitakis K Marmagkiolis K. Machine learning-augmented propensity score analysis of percutaneous coronary intervention in over 30 million cancer and non-cancer patients. Front Cardiovasc Med. (2021) 8:620857. 10.3389/fcvm.2021.620857 33889598 PMC8055825

[25] Charlson M Szatrowski TP Peterson J Gold J. Validation of a combined comorbidity index. J Clin Epidemiol. (1994) 47:1245–51. 10.1016/0895-4356(94)90129-5 7722560

[26] Ko T Higashitani M Uemura Y Utsunomiya M Yamaguchi T Matsui A. Clinical outcome and diverse risk factors for different therapeutic target locations of peripheral artery disease. J Atheroscler Thromb. (2020) 27:769–79. 10.5551/jat.52647 31723087 PMC7458788

[27] Barshes NR Sharath S Zamani N Smith K Serag H Rogers SO. Racial and geographic variation in leg amputations among Texans. Texas Public Health J. (2018) 70:22–7. 30873515 PMC6414040

[28] Allison MA Ho E Denenberg JO Langer RD Newman AB Fabsitz RR. Ethnic-specific prevalence of peripheral arterial disease in the united states. Am J Prev Med. (2007) 32:328–33. 10.1016/j.amepre.2006.12.010 17383564

[29] Shaw PM Chandra V Escobar GA Robbins N Rowe V Macsata R. Controversies and evidence for cardiovascular disease in the diverse Hispanic population. J Vasc Surg. (2018) 67:960–9. 10.1016/j.jvs.2017.06.111 28951154

[30] Hunt KJ Resendez RG Williams K Haffner SM Stern MP Hazuda HP. All-cause and cardiovascular mortality among Mexican-American and non-Hispanic white older participants in the san Antonio heart study— evidence against the “Hispanic paradox”. Am J Epidemiol. (2003) 158:1048–57. 10.1093/aje/kwg249 14630600

[31] Rutledge MS McLaughlin CG. Hispanics and health insurance coverage: the rising disparity. Med Care. (2008) 46:1086–92. 10.1097/MLR.0b013e31818828e3 18815531

[32] Fowkes FG Rudan D Rudan I Aboyans V Denenberg JO McDermott MM. Comparison of global estimates of prevalence and risk factors for peripheral artery disease in 2000 and 2010: a systematic review and analysis. Lancet. (2013) 382:1329–40. 10.1016/S0140-6736(13)61249-0 23915883

[33] Criqui MH Aboyans V. Epidemiology of peripheral artery disease. Circ Res. (2015) 116:1509–26. 10.1161/CIRCRESAHA.116.303849 25908725

[34] Criqui MH Langer RD Fronek A Feigelson HS Klauber MR McCann TJ. Mortality over a period of 10 years in patients with peripheral arterial disease. N Engl J Med. (1992) 326:381–6. 10.1056/NEJM199202063260605 1729621

[35] Kalbaugh CA Loehr L Wruck L Lund JL Matsushita K Bengtson LG. Frequency of care and mortality following an incident diagnosis of peripheral artery disease in the inpatient or outpatient setting: the aric (atherosclerosis risk in communities) study. J Am Heart Assoc. (2018) 7:e007332. 10.1161/JAHA.117.007332 29654201 PMC6015432

[36] Brothers TE Zhang J Mauldin PD Tonnessen BH Robison JG Vallabhaneni R. Better survival for African and Hispanic/Latino Americans after infrainguinal revascularization in the society for vascular surgery vascular quality initiative. J Vasc Surg. (2017) 65:1062–73. 10.1016/j.jvs.2016.10.105 28189358

[37] Fryer K Santos HP Jr. Pedersen C Stuebe AM. The Hispanic paradox: socioeconomic factors and race/ethnicity in breastfeeding outcomes. Breastfeed Med. (2018) 13:174–80. 10.1089/bfm.2017.0157 29485909 PMC5899276

[38] Olsen R Basu Roy S Tseng HK. The Hispanic health paradox for older Americans: an empirical note. Int J Health Econ Manage. (2019) 19:33–51. 10.1007/s10754-018-9241-4 29682677

[39] Bacon E Riosmena F Rogers RG. Does the Hispanic health advantage extend to better management of hypertension? The role of socioeconomic status, sociobehavioral factors, and health care access. Biodemogr Soc Biol. (2017) 63:262–77. 10.1080/19485565.2017.1353407 29035106 PMC5864248

[40] Rizzo JA Chen J Laurich C Santos A Martinsen BJ Ryan MP. Racial disparities in pad-related amputation rates among native Americans and non-Hispanic whites: An hcup analysis. J Health Care Poor Underserved. (2018) 29:782–800. 10.1353/hpu.2018.0058 29805140

[41] Rivero M Nader ND Blochle R Harris LM Dryjski ML Dosluoglu HH. Poorer limb salvage in African American men with chronic limb ischemia is due to advanced clinical stage and higher anatomic complexity at presentation. J Vasc Surg. (2016) 63:1318–24. 10.1016/j.jvs.2015.11.052 27005751

[42] Hong MS Beck AW Nelson PR. Emerging national trends in the management and outcomes of lower extremity peripheral arterial disease. Ann Vasc Surg. (2011) 25:44–54. 10.1016/j.avsg.2010.08.006 21172580

