# Supplementary material for: Disparities in Peripheral Artery Disease Hospitalizations Identified Among Understudied Race-Ethnicity Groups
Source: Front Cardiovasc Med. 2021 May 24;8:692236. doi: 10.3389/fcvm.2021.692236 (PMC8180581; doi:10.3389/fcvm.2021.692236)
Supplement: Supplementary file 1 [file Table_1.docx]

**Supplemental Table 1. ICD-9 codes**

| **Variables** | **ICD-9 Codes** |
| --- | --- |
| Critical Limb Ischemia | 440.22, 440.23, 440.24, 707.10, 707.11, 707.12, 707.13, 707.14, 707.15, 707.19, 785.4 |
| Peripheral Artery Disease | 440.2, 440.20, 440.21, 440.22, 440.23, 440.24, 440.29, 440.3, 440.0, 440.30, 440.31, 440.32, 440.9, 249.7, 249.71, 250.7, 250.71, 250.72, 250.73, 443.1, 443.81, 443.9, 444.22, 444.81, 785.4 |
| Major Amputation | 84.10, 84.13, 84.14, 84.15, 84.16, 84.17, 84.3 |

Abbreviations: ICD=the International Classification of Disease
